# Exercise training mitigates ER stress and UCP2 deficiency-associated coronary vascular dysfunction in atherosclerosis

**DOI:** 10.1038/s41598-021-94944-5

**Published:** 2021-07-29

**Authors:** Junyoung Hong, Eunkyung Park, Jonghae Lee, Yang Lee, Bridgette V. Rooney, Yoonjung Park

**Affiliations:** 1 Department of Health and Human Performance, Laboratory of Integrated Physiology, University of Houston, 3875 Holman St, Houston, TX 77204-6015 USA; 2 Department of Medical Physiology, College of Medicine, Texas A&M University, College Station, TX 77807 USA; 3 Geocontrol Systems Inc, NASA Johnson Space Center, Houston, TX 77058 USA

**Keywords:** Cardiovascular biology, Atherosclerosis, Coronary artery disease and stable angina

## Abstract

Endoplasmic reticulum (ER) stress and uncoupling protein-2 (UCP2) activation are opposing modulators of endothelial dysfunction in atherosclerosis. Exercise reduces atherosclerosis plaques and enhances endothelial function. Our aim was to understand how exercise affects ER stress and UCP2 activation, and how that relates to endothelial dysfunction in an atherosclerotic murine model. Wild type (C57BL/6, WT) and apolipoprotein-E-knockout (ApoE^tm1Unc^, ApoE KO) mice underwent treadmill exercise training (EX) or remained sedentary for 12 weeks. Acetylcholine (ACh)-induced endothelium-dependent vasodilation was determined in the presence of an eNOS inhibitor (L-NAME), UCP2 inhibitor (genipin), and ER stress inducer (tunicamycin). UCP2, ER stress markers and NLRP3 inflammasome signaling were quantified by western blotting. p67^phox^ and superoxide were visualized using immunofluorescence and DHE staining. Nitric oxide (NO) was measured by nitrate/nitrite assay. ACh-induced vasodilation was attenuated in coronary arterioles of ApoE KO mice but improved in ApoE KO-EX mice. Treatment of coronary arterioles with L-NAME, tunicamycin, and genipin significantly attenuated ACh-induced vasodilation in all mice except for ApoE KO mice. Exercise reduced expression of ER stress proteins, TXNIP/NLRP3 inflammasome signaling cascades, and Bax expression in the heart of ApoE KO-EX mice. Further, exercise diminished superoxide production and NADPH oxidase p67^phox^ expression in coronary arterioles while simultaneously increasing UCP2 expression and nitric oxide (NO) production in the heart of ApoE KO-EX mice. Routine exercise alleviates endothelial dysfunction in atherosclerotic coronary arterioles in an eNOS, UCP2, and ER stress signaling specific manner, and resulting in reduced TXNIP/NLRP3 inflammasome activity and oxidative stress.

## Introduction

Atherosclerosis is a major risk factor for cardiovascular disease and is one of the leading causes of death in the United States^1^. It is a chronic inflammatory disease of both small and large arteries and is characterized by large-scale lipid plaques in vessel walls^2^. These plaques are responsible for both mechanical and physiological reductions in endothelial function. Endothelial dysfunction is considered a hallmark indicator of the development and progression of atherosclerosis, and is associated with reductions in NO bioavailability along with increases in inflammation, reactive oxygen species (ROS) and apoptosis around the atherosclerotic lesion^3^.

Endoplasmic reticulum (ER) stress precipitates the initiation and progression of atherosclerosis and modulates endothelial dysfunction by influencing eNOS signaling pathway regulation^4^. Impaired ER homeostasis, due to many factors including hypercholesteremia and hyperlipidemia, interrupts normal protein folding in the ER lumen, resulting in aberrant protein conformations that trigger the unfolded protein response (UPR). The UPR is regulated by three crucial ER membrane proteins: PKR-like endoplasmic reticulum kinase (PERK), inositol-requiring enzyme 1 (IRE1), and activating transcription factor 6 (ATF6). Persistent UPR activity induces ER stress, which may trigger increases in oxidative stress, IRE1-mediated inflammation, and CCAAT-enhancer-binding protein homologous protein (CHOP)-mediated apoptosis signaling^4^.

Excessive reactive oxygen species (ROS) production that overwhelms the antioxidant defense systems is one of the major contributing factors for endothelial dysfunction, and may lead to damage of the vascular endothelium^5^ and results in significant reductions of NO bioavailability in atherosclerosis^6^. Uncoupling protein-2 (UCP2) is a mitochondrial inner membrane protein that serves as an important negative regulator of mitochondrial-derived ROS production^6^. Consequently, it has emerged as an important antioxidant that prevents the development of atherosclerosis^7^, as well as ameliorates endothelial dysfunction by increasing NO bioavailability in metabolic disorders^8^.

Recently, it has been reported that thioredoxin-interacting protein (TXNIP) is a key factor linking ER stress to oxidative stress and inflammation^9^. It is also associated with endothelial dysfunction and the development of atherosclerosis^10^. The nucleotide-binding oligomerization domain (NOD)-like receptor pyrin domain-containing 3 (NLRP3) inflammasome consists of a multimeric protein complex: NLRP3, apoptosis-associated speck-like protein containing (ASC), and caspase-1. The NLRP3 inflammasome is a pivotal regulator of initial immune responses^11^ and is activated by TXNIP in response to ER stress^12^. Activation of the inflammasome promotes the maturation and secretion of both IL-1β and IL-18 in a caspase-1 dependent manner^13^. Each of these cytokines, as well as activation of the NLRP3 inflammasome itself, has been implicated in endothelial dysfunction^13,14^ and atherosclerosis pathogenesis^15^. However, little is known about the mechanistic link between ER stress, TXNIP/NLRP3 inflammasome activation, and UCP2 activity on coronary vascular dysfunction in atherosclerosis.

The cardiovascular benefits of exercise have been well documented to include improvements in endothelial function, and reductions in both inflammation and oxidative stress in atherosclerosis^16^. A handful of studies have attributed the benefit of routine exercise to the reduction in ER stress by highlighting that ER stress is associated with endothelial dysfunction in diabetes^17^ and atherosclerosis^18^. Additionally, previous studies from our lab have determined that vascular function is improved by routine exercise due to UCP2-mediation in atherosclerosis^18^ and decreases of NLRP3 inflammasome activity in obesity^19^. However, no study to date has sought to determine the combined interaction of ER stress, UCP2 expression, and NLRP3 activity in response to exercise in a murine atherosclerotic model.

Therefore, the main purpose of this study was to determine the possible underlying mechanisms responsible for the effect of exercise on ER stress and UCP2 deficiency-associated coronary vascular dysfunction in atherosclerosis. To answer these questions, we first evaluated whether ACh-induced vasodilation is impaired in coronary arterioles in atherosclerosis, and then we determined whether exercise training could improve coronary function by modulating the ER stress and UCP2 pathways, including the TXNIP/NLRP3 inflammasome cascade and oxidative stress response in atherosclerosis. We hypothesized that exercise training would mitigate endothelial dysfunction in atherosclerotic coronary arterioles through elevation of NO bioavailability. Also, we hypothesized that exercise training would alleviate ER stress-associated endothelial dysfunction in atherosclerotic coronary arterioles by reducing ER stress signaling and its downstream cascade including TXNIP/NLRP3 inflammasome. We further hypothesized that exercise training would mitigate UCP2 deficiency-associated vascular dysfunction through the restoration of UCP2 expression and reduction of oxidative stress in coronary arterioles of atherosclerosis.

## Results

### Animal and vascular characteristics

The animal characteristics have been presented in our previous study^18^. Neither initial nor maximal intraluminal diameters of coronary arterioles were significantly different in WT and atherosclerotic ApoE KO, and exercise training did not affect the diameters in either group (Table 1).

**Table 1:** Vessel characteristics.

	Sedentary		Exercise	
	WT	ApoE KO	WT-EX	ApoE KO-EX
N	10	9	6	7
Coronary arterioles initial lumen diameter, µm	81.88 ± 6.04	75.70 ± 11.33	95.53 ± 6.70	91.78 ± 8.24
Coronary arterioles maximal lumen diameter, µm	125.89 ± 11.31	116.99 ± 8.87	126.14 ± 7.68	119.79 ± 7.79

The initial lumen diameter and maximal lumen diameter of coronary arterioles were measured at 24–25 weeks of age.

### Exercise training ameliorates endothelial dysfunction via an increase in NO concentration in coronary arterioles of ApoE KO mice

**Figure 1:**
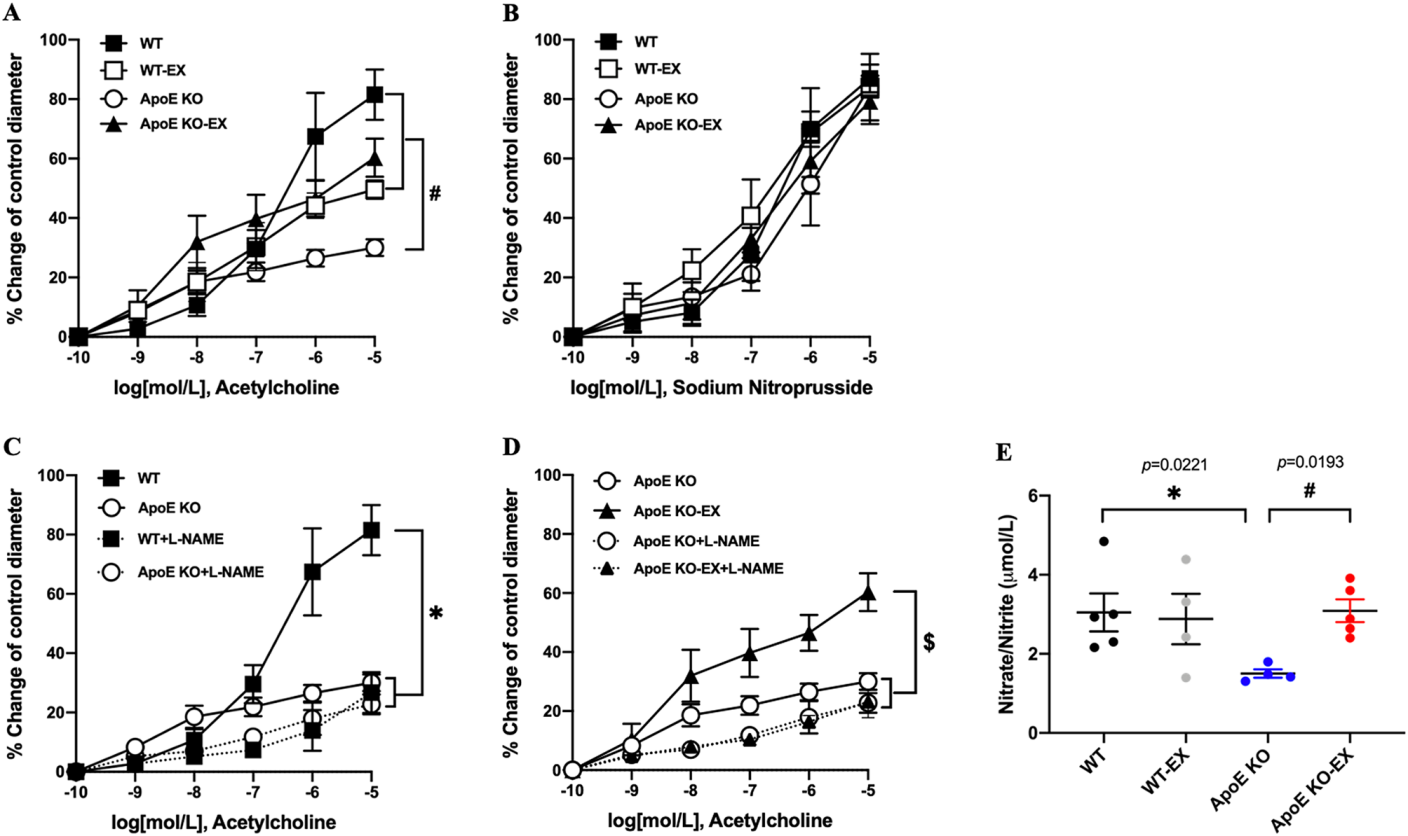
Effect of exercise training on endothelial dysfunction and NO production in ApoE KO mice. (**A**): Isolated coronary arterioles from WT (n = 4), WT-EX (n = 4), ApoE KO (n = 5), and ApoE KO-EX (n = 4) were measured the response of ACh in a dose-dependent manner. (**B**): Endothelium-independent vasodilation was measured in isolated coronary arterioles from WT + SNP (n = 5), WT-EX + SNP (n = 4), ApoE KO + SNP (n = 5), ApoE KO-EX + SNP (n = 4). (**C**,**D**): ACh-induced vasodilation in coronary arterioles was measured in the presence of L-NAME, the eNOS inhibitor, from WT + L-NAME (n = 4), WT-EX + L-NAME (n = 4), ApoE KO + L-NAME (n = 5), and ApoE KO-EX + L-NAME (n = 4). (**E**): Total nitrate/nitrite concentration was analyzed in mice heart from each group (n = 4–5/groups). Values are means ± SEM. ^*^p < 0.05 versus WT, ^#^*p* < 0.05 versus ApoE KO, ^§^*p* < 0.05 versus ApoE KO-EX.

ACh-induced endothelium-dependent vasodilation was significantly attenuated in coronary arterioles in ApoE KO mice compared with WT mice (*p* < 0.05; Fig. 1A). However, exercise training significantly improved ACh-induced vasodilation in coronary arterioles of ApoE KO-EX mice compared to ApoE KO mice (*p* < 0.05; Fig. 1A). Figure 1B shows that the SNP-induced vasodilatory response was identical in the coronary arterioles of all mouse groups (*p* > 0.05).

Treatment of isolated coronary arterioles with L-NAME significantly attenuated vasodilation in response to ACh in the WT and ApoE KO-EX mice (*p* < 0.05), but not in ApoE KO mice (Fig. 1C,D). Furthermore, the absolute concentration of total nitrate/nitrite levels was significantly reduced in the heart of ApoE KO compared with WT (*p* < 0.05), but its level was significantly elevated in response to exercise training in the heart of ApoE KO-EX mice compared with ApoE KO mice (Fig. 1E).

### Exercise training mitigates ER stress-associated coronary vascular dysfunction in ApoE KO mice

**Figure 2:**
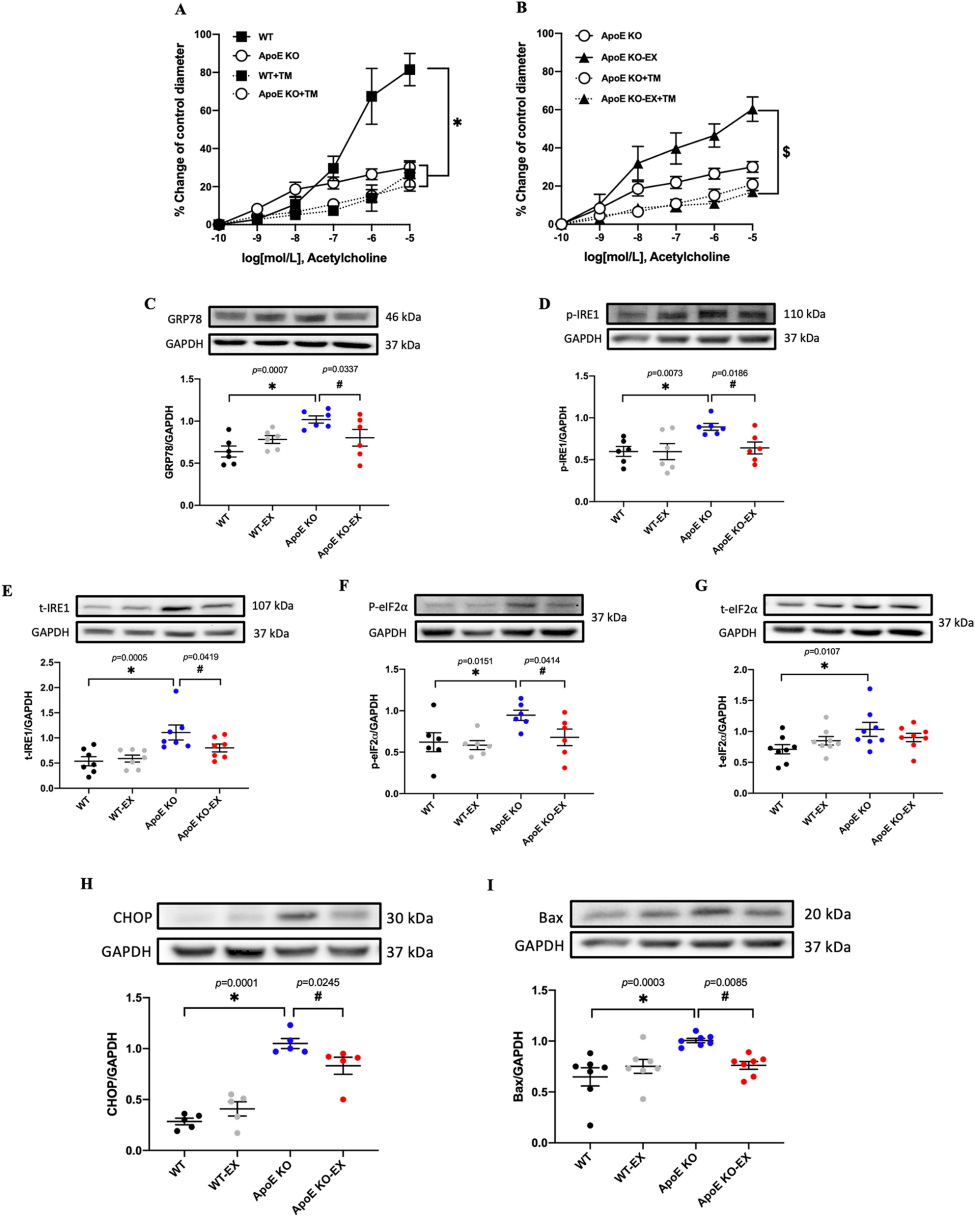
Effect of exercise training on ER stress-associated endothelial dysfunction in coronary arterioles and the expression of ER stress and apoptosis in the heart of ApoE KO mice. (**A**–**B**): ACh-induced vasodilation was measured in the presence of ER stress inducer (tunicamycin) from the isolated coronary arterioles in WT + TM (n = 4), WT-EX + TM (n = 4), ApoE KO + TM (n = 5), and ApoE KO-EX + TM (n = 4). (**C**–**H**): The protein expression of ER stress markers; GRP78 (**C**; n = 6/groups), p-IRE1 (**D**; n = 6/groups), t-IRE1 (**E**; n = 7/groups), p-eIF2α (**F**; n = 6/groups), t-eIF2α (**G**; n = 8/groups), CHOP (**H**; n = 5/groups), and pro-apoptotic marker, Bax (**I**; n = 7/groups) were analyzed by western blot. Values are presented as means ± SEM. ^*^p < 0.05 versus WT, ^#^*p* < 0.05 versus ApoE KO, ^§^*p* < 0.05 versus ApoE KO-EX.

Figure 2A,B show that incubation with ER stress inducer (Tunicamycin) significantly impaired ACh-induced vasodilation in coronary arterioles in both WT and ApoE KO-EX mice (*p* < 0.05), whereas no further deterioration was shown in coronary arterioles in ApoE KO mice. Likewise, the expression of ER stress markers including GRP78, p/t-IRE1, p/t-eIF2α, and CHOP were significantly increased in the heart of ApoE KO mice compared with WT mice (*p* < 0.05; Fig. 2C–H), but up-regulated expression of these ER stress markers were significantly reduced by exercise training in the heart of ApoE KO-EX mice (*p* < 0.05; Fig. 2C–H). Finally, the elevated expression of the pro-apoptotic marker Bax was also significantly diminished in the heart of ApoE KO mice in response to exercise training (*p* < 0.05; Fig. 2I).

### Exercise training reduces the expression of TXNIP/NLRP3 inflammasome in the heart of ApoE KO mice

**Figure 3:**
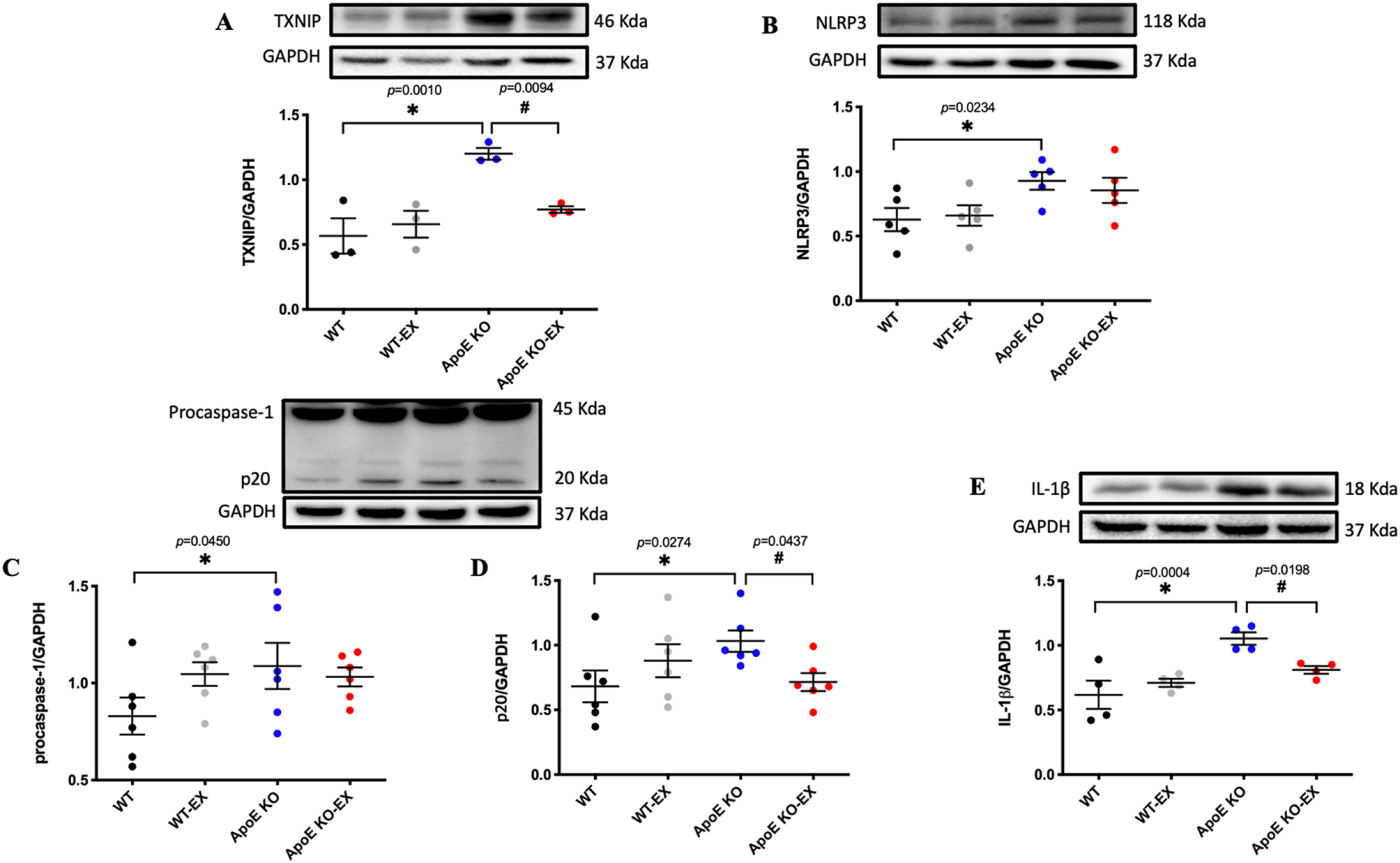
Effect of exercise training on TXNIP/NLRP3 inflammasome in the heart of ApoE KO mice. (**A**–**E**): The protein expression of TXNIP (**A**; n = 3/groups), NLRP3 (**B**; n = 5/groups), pro-caspase-1 (**C**; n = 6/groups), caspase-1 p20 (**D**; n = 6/groups), and IL-1β (**E**; n = 4/groups) were measured by immunoblot analysis from each group. Bar graph values are presented as means ± SEM. ^*^p < 0.05 versus WT, ^#^*p* < 0.05 versus ApoE KO.

The expression of TXNIP, NLRP3, pro-caspase-1, caspase-1 p20, and IL-1β were significantly elevated in the heart of ApoE KO mice compared with WT mice, and exercise training significantly reduced the expression of TXNIP, caspase-1 p20, and IL-1β expression in the heart of ApoE KO-EX mice (*p* < 0.05; Fig. 3A–E).

### Exercise training mitigates UCP2 deficiency-mediated endothelial dysfunction in coronary arterioles and oxidative stress in heart of ApoE KO mice

**Figure 4:**
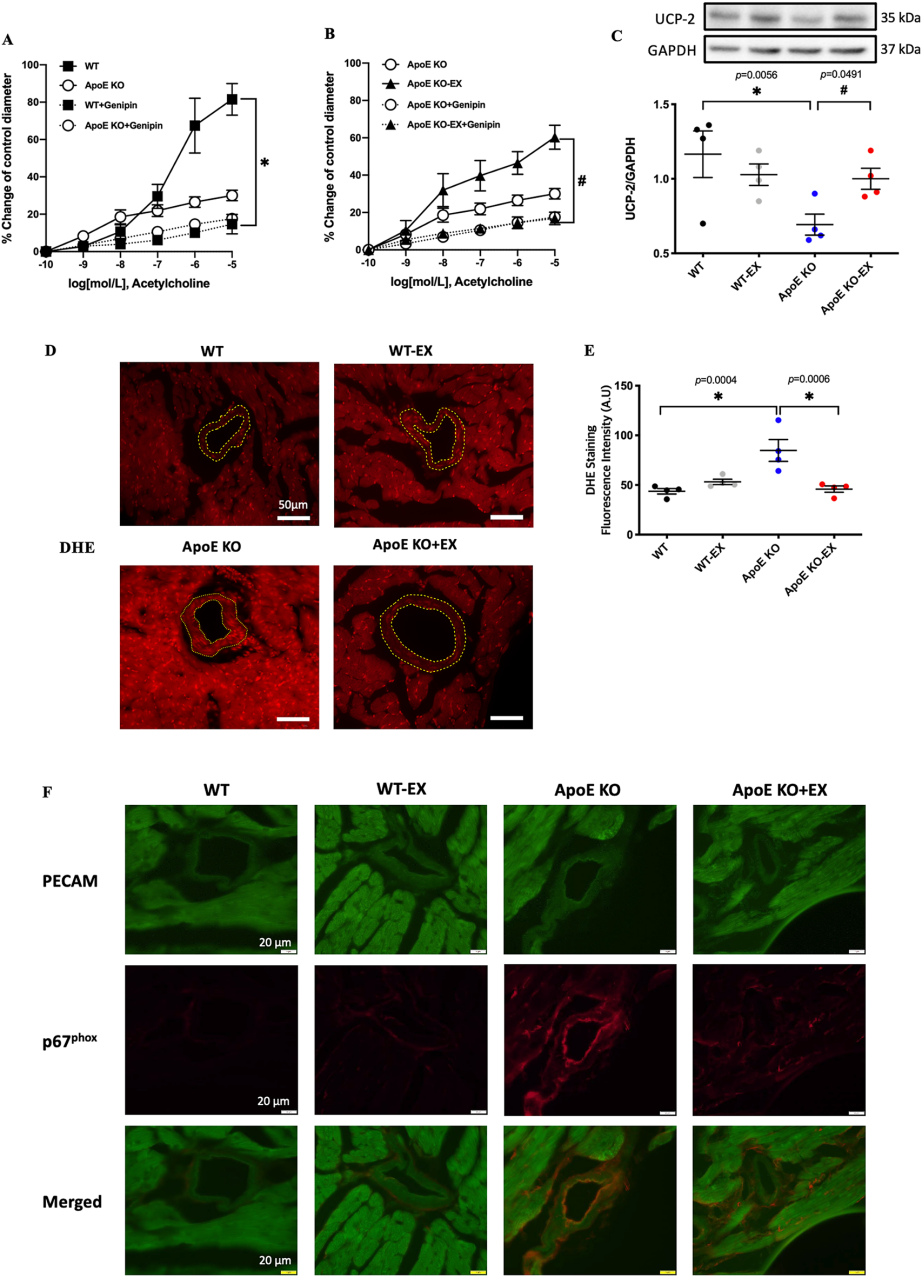
Effect of exercise training on UCP2 deficiency-mediated endothelial dysfunction in coronary arterioles and oxidative stress in the heart of ApoE KO mice. (**A,B**): ACh-induced vasodilation was measured in the present of UCP2 inhibitor (genipin) from isolated coronary arterioles in WT + genipin (n = 4), WT-EX + genipin (n = 4), ApoE KO + genipin (n = 5) and ApoE KO-EX + genipin (n = 4). (**C**): The protein expression of UCP2 was measured by immunoblot analysis from each group (n = 4/groups). (**D**): Representative images of DHE staining from each group. Magnification: × 20. Scale bar = 50 μm. (**E**): Quantified DHE intensity from four independent experiments using the different samples (n = 4/groups). (**F**): Immunofluorescence image of NADPH oxidase subunit p67phox in the coronary arterioles. Magnification: × 40. Scale bar = 20 μm. Values are presented as means ± SEM. ^*^p < 0.05 versus WT, ^#^*p* < 0.05 versus ApoE KO.

Figure 4A,B show that treatment with UCP2 inhibitor (genipin) significantly attenuated ACh-induced vasodilation in coronary arterioles in both WT and ApoE KO-EX, but no further impairment was shown in coronary arterioles in ApoE KO mice. Thus, protein expression of UCP2 was significantly reduced in the heart of ApoE KO mice compared to WT mice, but its expression was increased by exercise training (*p* < 0.05; Fig. 4C).

The O_2_^−^ production was increased in coronary arterioles of ApoE KO heart compared with WT, but was diminished by exercise training shown by DHE staining (*p* < 0.05; Fig. 4D,E). Immunofluorescence images demonstrated that NADPH oxidase subunit p67^phox^ was abundantly expressed in endothelial cells of coronary arterioles in ApoE KO mice, and its expression appeared to be reduced by exercise training in ApoE KO-EX (Fig. 4F).

## Discussion

Our results demonstrate that the cardiovascular benefits of exercise are intimately linked to the relationship between ER stress, UCP2, NLRP3 inflammasome, and oxidative stress. Specifically, our results indicate that exercise training improved endothelial function in coronary arterioles of ApoE KO mice through NO-dependent signaling pathways. We further showed that exercise training ameliorated ER stress-associated endothelial dysfunction in coronary arterioles in ApoE KO mice by reducing ER stress and TXNIP/NLRP3 inflammasome signaling. Lastly, we determined that exercise training rescued UCP2 deficiency-mediated endothelial dysfunction in coronary arterioles of ApoE KO via down-regulation of ROS by means of up-regulating UCP2 expressions.

Exercise training has emerged as a potentially significant therapeutic strategy for the prevention and treatment of atherosclerosis^20^. These positive changes are directly linked to the amelioration of endothelial dysfunction by the preservation of NO bioavailability^21,22^. We reported in the previous study that exercise training reduced atherosclerotic plaque formation in the aorta from ApoE KO mice model^18^. More importantly, the current study showed that exercise training improved the ACh-induced endothelial function in coronary arterioles of ApoE KO mice (Fig. 1A–E) through endothelium-dependent NO signaling pathways. These NO-mediated beneficial effects of exercise on vascular function in atherosclerosis have been well-matched with a number of the previous studies^21–23^. However, our nitrite/nitrate assay data has a limitation to determine NO bioavailability. The method is an indirect quantitative determination of NO concentration by measuring total nitrite and nitrate concentration.

ER stress is a crucial factor for modulating endothelial dysfunction by regulation of oxidative stress, inflammation, and NO bioavailability in atherosclerosis^4^. Exercise training has been shown to be an effective therapeutic intervention for reducing ER stress burden in a variety of disease models^24^. Recently, vascular functional studies have shown that ER stress is a critical player in endothelial dysfunction by reducing eNOS expression and NO bioavailability in ApoE KO mice and other pathological animal models^17,18,25,26^. Our vascular functional data confirmed that ER stress induction directly impaired endothelial function in coronary arterioles of WT and ApoE KO-EX mice, but no further impairment was shown in ApoE KO mice (Fig. 2A,B). Also, exercise training effectively suppressed the elevated ER stress markers (Fig. 2C–H). Furthermore, exercise training positively counteracted the expression of the CHOP-mediated pro-apoptosis marker, Bax (Fig. 2I). Our findings are validated by the literature which shows that regular exercise training ameliorates ER stress-mediated endothelial dysfunction through the down-regulation of ER stress markers in mesenteric arteries from diabetic^17^ and ApoE KO mice^18^. Recent studies reported that the absence of peroxisome proliferator-activated receptor-δ (PPAR-δ) and PDE 5 abolished the exercise-induced beneficial changes to ER stress and NO-mediated vasodilatory signaling pathways in diabetes and aged animal models^17,27^. It suggested that exercise training reduces ER stress burden and increases NO bioavailability via the regulation of PPAR-δ and cGMP/PKG/PDE5 signaling. Collectively, we believe our current study may support a causal link between exercise training and improved NO-mediated endothelial function through the down-regulation of ER stress in ApoE KO mice (Fig. 2). This might occur through the improvement of PPAR-δ and cGMP/PKG/PDE5 signaling cascades, which in turn, activate the reduction of the ER stress, improve vasorelaxation by elevation of NO concentration, and induces cellular survival signaling. Furthermore, a treatment of ER stress inhibitor would demonstrate the solid causal link between ER stress and endothelial impairment in coronary arterioles in ApoE KO mice and it should be addressed in the future study.

The ER stress-induced inflammatory response in endothelial cells is mediated through the activation of the TXNIP/NLRP3 inflammasome signaling cascade^28^. The TXNIP/NLRP3 inflammasome signaling is highly associated with the initiation and progression of atherosclerosis^29^. Previous vascular functional studies confirmed that NLRP3 inflammasome plays a pivotal role in the regulation of endothelial dysfunction by activation of caspase-1, IL-1β, and IL-18 in metabolic disease models^18,30,31^. Again, exercise is an established intervention for inflammation-induced vascular dysfunction by promoting anti-inflammatory milieu in cardiovascular disease^32^, however, very little data exists regarding the effect of exercise on TXNIP/NLRP3 inflammasome signaling in atherosclerosis. Our findings show that exercise training reduces the expression of TXNIP and caspase-1 p20, IL-1β in ApoE KO (Fig. 3A–E). Also, exercise training reduces the elevated expression of TXNIP, NLRP3, and its downstream cascade in obese mice model^19^ and in the Aβ-injected mice^33^. In further support, the blocking of NLRP3 restores eNOS expression and NO production by reducing expression of NLRP3, and thereby eliminating the downstream products of its activation in ApoE KO^34^ and diabetic mouse models^35^. Collectively, our current findings suggest that the elimination of TXNIP/NLRP3 inflammasome signaling by exercise might be a key mechanism for improving coronary endothelial function along with ER stress. However, further studies are necessary to investigate the causal relationship between exercise training and ER stress-induced TXNIP/NLRP3 inflammasome signaling in coronary arterioles in atherosclerosis.

UCP2 is one of the major antioxidant proteins dampening mitochondria-derived ROS production^5^, in turn, regulating NO bioavailability. The deficiency of UCP2 is associated with the initiation and progression of atherosclerosis^7^ and endothelial dysfunction with increased ROS and reduced NO expression^6,36^. The beneficial effect of exercise on the regulation of UCP2 expression and oxidative stress is well described in peripheral blood mononuclear cells (PBMCs) and in the aorta of aged rats^37^. However, there is very little data regarding how exercise influences UCP2 signaling in atherosclerosis. Our vascular functional data show that the treatment of UCP2 inhibitor, genipin, revoked the exercise benefit they gained in ApoE KO-EX, and the reduced protein expression of UCP2 was increased by exercise training (Fig. 4). Our findings suggest that exercise training could attenuate UCP2 deficiency-mediated endothelial dysfunction possibly through the elevation of UCP2 expression. The elevation of UCP2 expression by exercise training occurs through the PGC1α/PPAR-δ pathway, which regulates ROS generation and increases eNOS expression^38–40^. Also, the impaired endothelial function was attenuated by up-regulation of UCP2 along with the elevation of eNOS expression/NO bioavailability and the reduction of ROS in human aortic endothelial cells (HAECs) and rat aorta^41^. It suggested that the activation PGC1α/PPAR-δ pathway by exercise increases UCP2 expression, which in turn, reduces ROS generation and elevates NO bioavailability. Likewise, exercise reduced the elevated superoxide production and NADPH oxidase expression in the coronary arterioles of ApoE KO mice (Fig. 4D–F). Accordingly, our findings suggest that the upregulation of UCP2 expression by exercise training ameliorates vascular dysfunction through the reduction of UCP2 deficiency-derived ROS production in atherosclerosis and it might be regulated by PGC1α/PPAR-δ signaling.

The current study can provide a more comprehensive understanding of the beneficial effect of exercise on cardiovascular diseases in atherosclerosis. We have reported the protective effect of exercise training on ER stress-mediated endothelial dysfunction in mesenteric arteries in atherosclerosis^18^. In addition, here we report that exercise training ameliorates ER stress and UCP2 deficiency-associated endothelial dysfunction in different vascular beds, coronary arterioles where mainly regulate blood flow to the cardiac muscle, in atherosclerosis. Also, we determine more in-depth underlying molecular mechanisms by which exercise training improves coronary endothelial function by regulating ER stress, TXNIP/NLRP3 inflammasome signaling cascades, and UCP2-regulated ROS generation in atherosclerosis. In conclusion, our study reinforces that exercise training is a profoundly effective therapy for the treatment and prevention on ER stress and UCP2 deficiency-associated coronary arteriole dysfunction in atherosclerosis via regulation of inflammation and oxidative stress including TXNIP/NLRP3 inflammasome and UCP2 deficiency-mediated ROS generation. However, further causal mechanistic studies are required to decisively establish how exercise influences these changes.

## Methods

### Animal models

Five-six week old male wild type (C57BL/6, WT) and apolipoprotein knock out (ApoE^tm1Unc^, ApoE KO) mice were supplied from the Jackson Laboratory (Bar Harbor, ME). WT and ApoE KO mice were randomly divided into either exercise training (EX) or sedentary groups. Mice were housed in a temperature-controlled (22–23 °C) animal facility with 12 h of light/dark cycles and allowed free access to water and chow. WT and WT-EX mice were fed a normal chow and ApoE KO and ApoE KO-EX mice were fed a western atherogenic diet (0.2% cholesterol, 42% Kcal from fat, TD.88137, ENVIGO) for 17 weeks to accelerate atherosclerosis development. All mice were sacrificed at 24–25 weeks old. All experimental protocols were approved by the Institutional Animal Care and Use Committee at the University of Houston (15-020). The described all experiments on animals were performed in agreement with the NIH Guidelines for the Use of Animals in Research and were carried out in compliance with the ARRIVE guidelines.

### Exercise training protocols

The exercise training protocol was previously described in detail^42^. At 7–8 weeks of age, WT-EX and ApoE KO-EX mice were subjected to five consecutive days of aerobic exercise on a motorized rodent treadmill (Columbus Instruments, Columbus, OH). Preceding the exercise intervention, the EX mice were acclimated to running on a treadmill for 1 week. Each exercise training session consisted of 1 h of running on a motor-driven treadmill at 15 m/min at a 5° grade (60 ~ 80% of VO_2_ max), 5 days/week for 15–16 weeks. The programmed exercise protocol consisted of warm-up (5 min), running (50 min), and cool-down (5 min). Sedentary groups of mice remained in the same room and conditions throughout the experiment. At 24–25 weeks of age, all mice were sacrificed by CO_2_ inhalation and bilateral thoracotomy 24 h after the final exercise training session.

### Functional assessment of isolated coronary arterioles of ApoE KO mice

After euthanasia, the heart was removed and rapidly placed in cold (4 °C) physiological saline solution (PSS). An isolated coronary arteriole (60–100 μm in internal diameter and 0.5–1.0 mm in length) was cannulated with glass micropipettes filled with PSS-albumin solution, and secured with surgical nylon sutures. The chamber was transferred to a stage of an inverted microscope (Nikon Eclipse Ti-S) and the cannulated arteriole was pressurized to 60cmH_2_O intraluminal pressure without the flow. After developing a stable basal tone (1 h), the vasodilation function was tested by different stimuli, as previously described^43^. The dose–dependent-diameter changes were established with an endothelium-dependent vasodilator, ACh (0.1 nmol/L to 10 µmol/L) in coronary arterioles. Tunicamycin (ER stress inducer, TM, 10 µmol/L), and genipin (UCP-2 inhibitor, 10 µmol/L) were each incubated with the isolated vessel for 20–30 min before measuring ACh-induced endothelium-dependent vasodilation. The contribution of NO to vasodilation was assessed by incubating the vessels with the *N*^*G*^-nitro-L-arginine methyl ester (L-NAME; NO synthases inhibitor; 10 μmol/L, 20 min). TM was dissolved in 0.1% DMSO and all other drugs were dissolved in PSS and then extraluminally administrated into the PSS in the chamber before measuring the vascular function. At the end of each experiment, the endothelium-independent vasodilator; sodium nitroprusside (SNP, 0.1 nmol/L to 10 µmol/L) was applied to the vessel to obtain smooth muscle-dependent vasodilation and then the vessel was exposed to 100 μmol/L SNP to obtain its maximal diameter at 60 cmH_2_O intraluminal pressure. These assessments were accomplished in coronary arterioles from animals assigned to all four experimental groups.

### Measurement of intracellular superoxide (O_2_^−^)

To evaluate the production of superoxide in coronary arterioles, the oxidative fluorescent dye dihydroethidium (DHE) was used. Frozen cardiac tissue embedded in OCT compound was sectioned at 10 µm thickness at − 25 °C using a cryostat (Leica CM 1950, Leica Biosystems Inc., Buffalo Grove, IL). The sections were placed on microscope slides, and the slides were dried at RT for 15 min. The slides in PBS were then incubated in a light-protected humidified incubator at 37 °C with 95% O_2_ and 5% CO_2_ for 1 h, after which the slides were incubated with 5 μm DHE for 15 min at RT in a dark room. Finally, the slides were washed 3 times in cold PBS (every 5 min) and were mounted with a cover slide in the darkroom. DHE stained images were visualized by an Olympus BX51 fluorescence microscope and acquired with 20X objective lens at an excitation peak of 545 nm with an emission spectral peak of 610 nm. Fluorescence intensity was measured by Image J.

### Measurement of total nitrate/nitrite concentration in heart tissue

Myocardial nitrate/nitrite concentration was estimated using a Colorimetric Assay Kit (78001, Cayman Chemical, Ahn Arbor, MI), as per the manufacturer’s instruction. To determine the total nitrate/nitrite level in the supernatant solution, the samples were duplicated and measured by a colorimetric microplate reader at 540 nm.

### Immunoblotting

Heart tissue was homogenized and lysed in RIPA buffer with protease/phosphatase inhibitor cocktails, and then centrifuged 15,000 g for 20 min at 4 °C and protein concentration was measured by BCA assay. Proteins from heart and cell lysates (30 μg per sample) were resolved by Tris-glycine sodium dodecyl sulphate-polyacrylamide (SDS-PAGE) gel electrophoresis and transferred to polyvinylidene difluoride (PVDF) membrane. Subsequently, the membrane was blocked with 5% non-fat milk in Tris-buffered saline-Tween 20 (0.05% TBST) for 30 min at RT and incubated overnight with respective primary antibodies. The primary antibodies were purchased from the following sources: TXNIP (Santa Cruz; sc-271237), NLRP3 (Abcam; ab4207), caspase-1 (Abcam; ab108362), IL-1β (Abcam; ab9722), p-IRE1 (Abcam; ab48187), IRE1 (Abcam; ab37073), p-eIF2α (Invitrogen; 44728G), eIF2α (Cell Signaling; 9722), CHOP (Invitrogen; MA1-250), Bax (Cell Signaling; 2772), UCP-2 (Santa Cruz; sc-390189), and GAPDH (Cell Signaling; sc47778). The membrane was then washed three times in TBST and incubated with horseradish peroxidase (HRP)-conjugated secondary antibodies for two hours at RT followed by washing three times with TBST. The blot bands were developed with enhanced chemiluminescence (Thermo Fisher Scientific Inc., MA, USA) for visualization, detected by ChemiDoc™ MP imager (Bio-Rad, CA), and quantified using NIH ImageJ software for densitometric analysis. Protein expression was normalized to the internal control, GAPDH. The full-length of the membrane was not able to provide because the blot was cut prior to incubating primary antibodies. All blots with membrane edges are provided in the Supplementary file.

### Immunofluorescence

Immunofluorescence experiments were performed as previously described with some modifications^19,44^ in order to determine p67^phox^ localization on the endothelium of coronary arterioles with diameters ranging from 50 to 100 μm. In brief, the freshly frozen heart was sectioned at 10 μm. After 1 h of acetone permeabilization, slides were incubated with a 10% goat serum in PBS for 30 min at RT after three washes in PBS. A mouse on mouse (Vector Laboratories) kit was used to prevent non-specific binding between testing tissue and an antibody from mouse host. The sections were then incubated for one hour at RT in a blocking buffer with primary antibodies, including PECAM1 (Abcam) and anti-p67^phox^ (BD Biosciences). After three washes in PBS, sections were incubated with the fluorescence-conjugated secondary antibody, Alexa Fluor 488 and Alexa Fluor 594 (Thermo Fischer Scientific) and mounted with ProloLong antifade solution. Negative controls were performed with the use of mouse IgG_2b_ and rabbit IgG (Santa Cruz Biotechnology). Images were taken with an Olympus BX41 fluorescence microscope, using 40 × objective. Microscope and camera settings were consistent throughout the process for continuity of data for all groups of samples.

### Data analysis

All data are presented as mean ± SEM. All diameter changes to pharmacological agonists were normalized to the control diameter. Statistical comparisons of vasoreactivity responses between groups were performed with two-way analysis of variance (ANOVA) for repeated measure and intergroup differences were tested with Fisher’s protected LSD test. The significance of intergroup differences observed in body weight, vessel diameters, and protein expressions were analyzed with one-way analysis of variance (one-way ANOVA) using software SPSS 22.0. Significance was accepted at *p* < 0.05.

## Supplementary Information

Supplementary Information.

## Data Availability

The data that supports the findings of this study are available from the corresponding author upon reasonable request.
