# Supplementary material for: Exercise training mitigates ER stress and UCP2 deficiency-associated coronary vascular dysfunction in atherosclerosis
Source: Sci Rep. 2021 Jul 29;11:15449. doi: 10.1038/s41598-021-94944-5 (PMC8322067; doi:10.1038/s41598-021-94944-5)

Exercise training mitigates ER stress and UCP2-associated coronary vascular dysfunction in atherosclerosis

Junyoung Hong[1], Eunkyung Park[1], Jonghae Lee[1], Yang Lee[2], Bridgette V. Rooney[1, 3] and Yoonjung Park[1]

Supplementary Data- Representative Western blot images

Figure 2C- GRP78

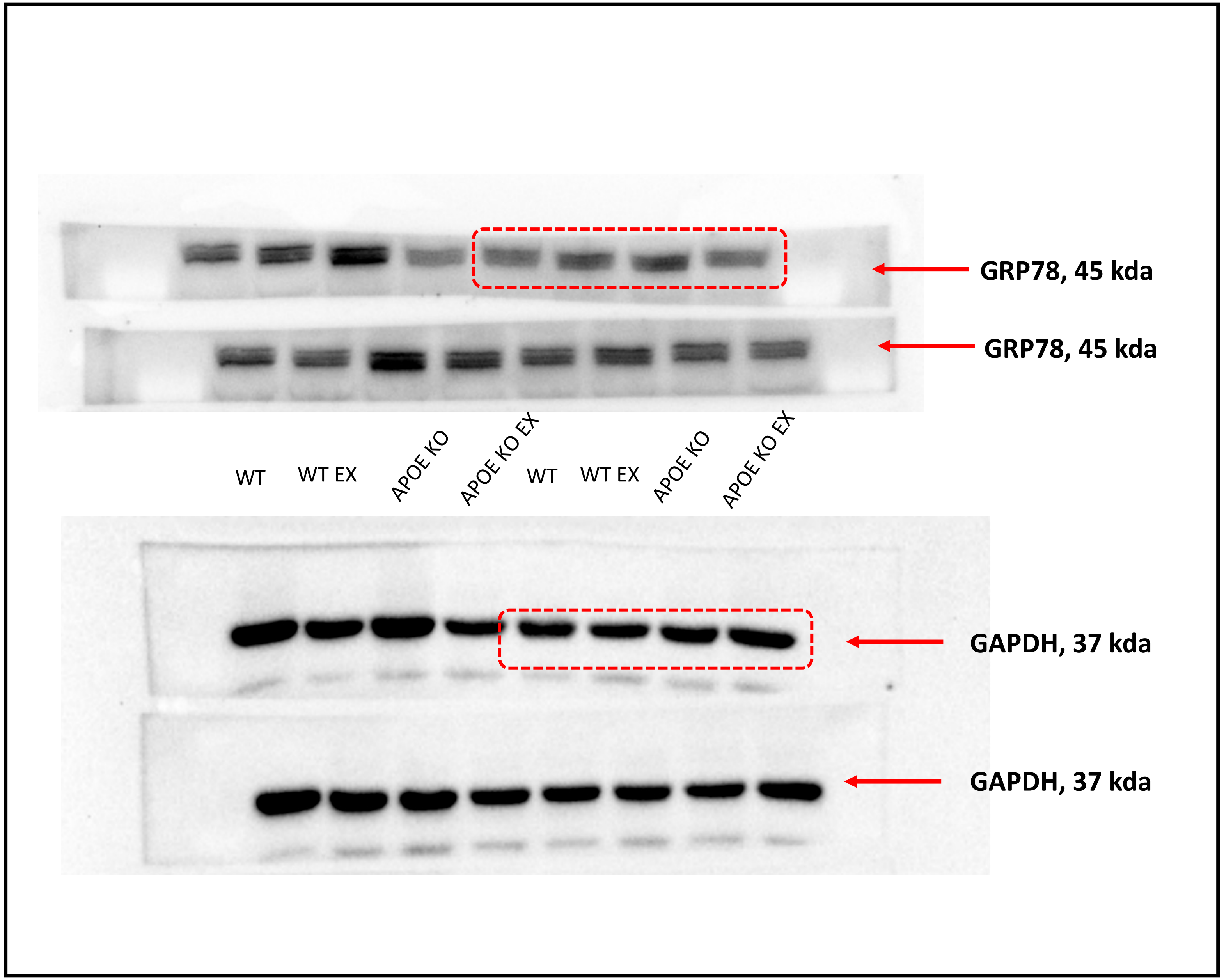

Supplementary Data
Representative Western blot images

Figure 2D & E- p-IRE1 & t-IRE1

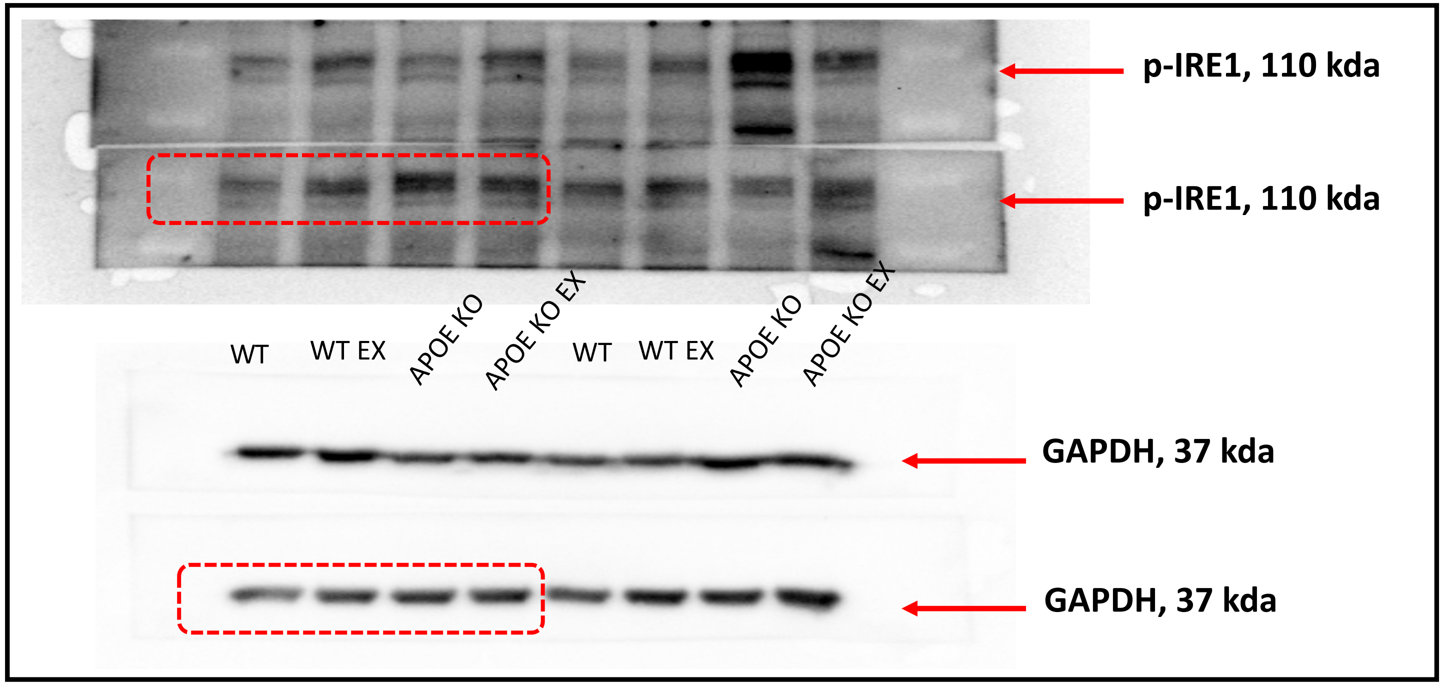

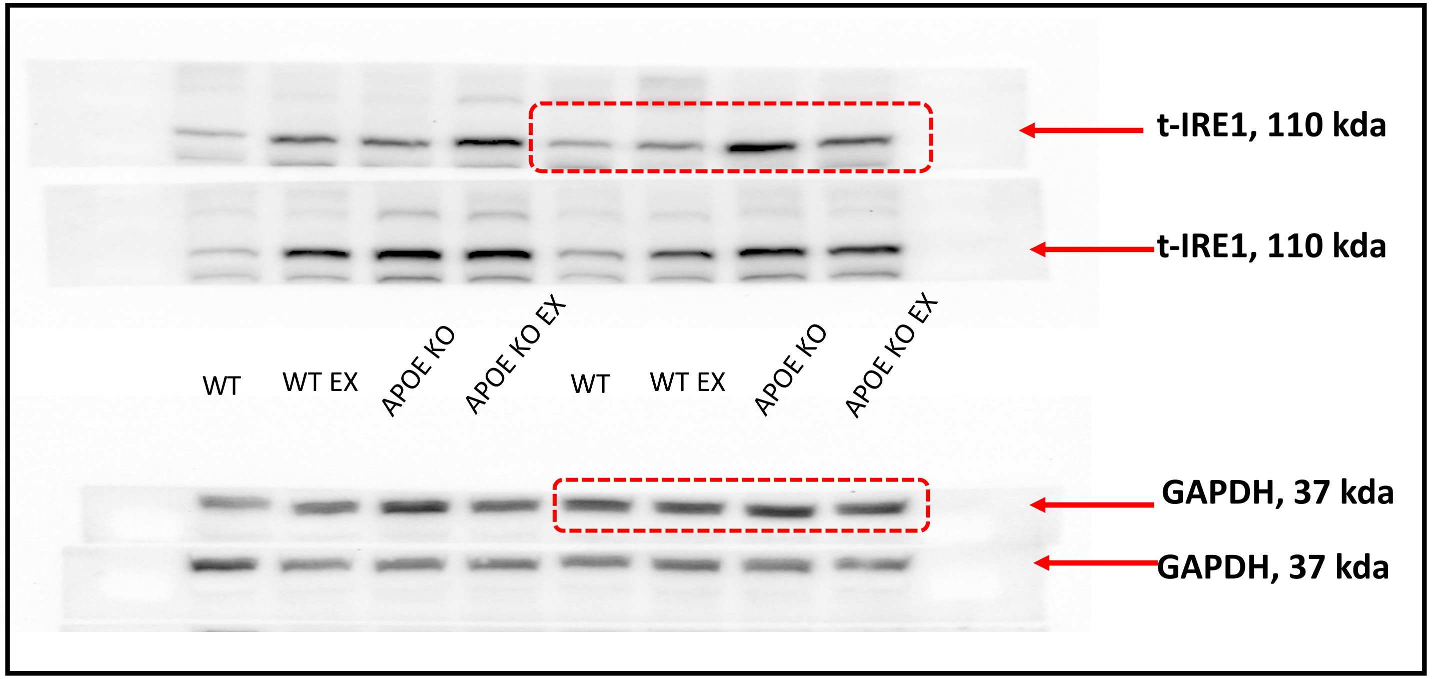

Supplementary Data
Representative Western blot images

Figure 2F & G- p-eIF2α & t-eIF2α

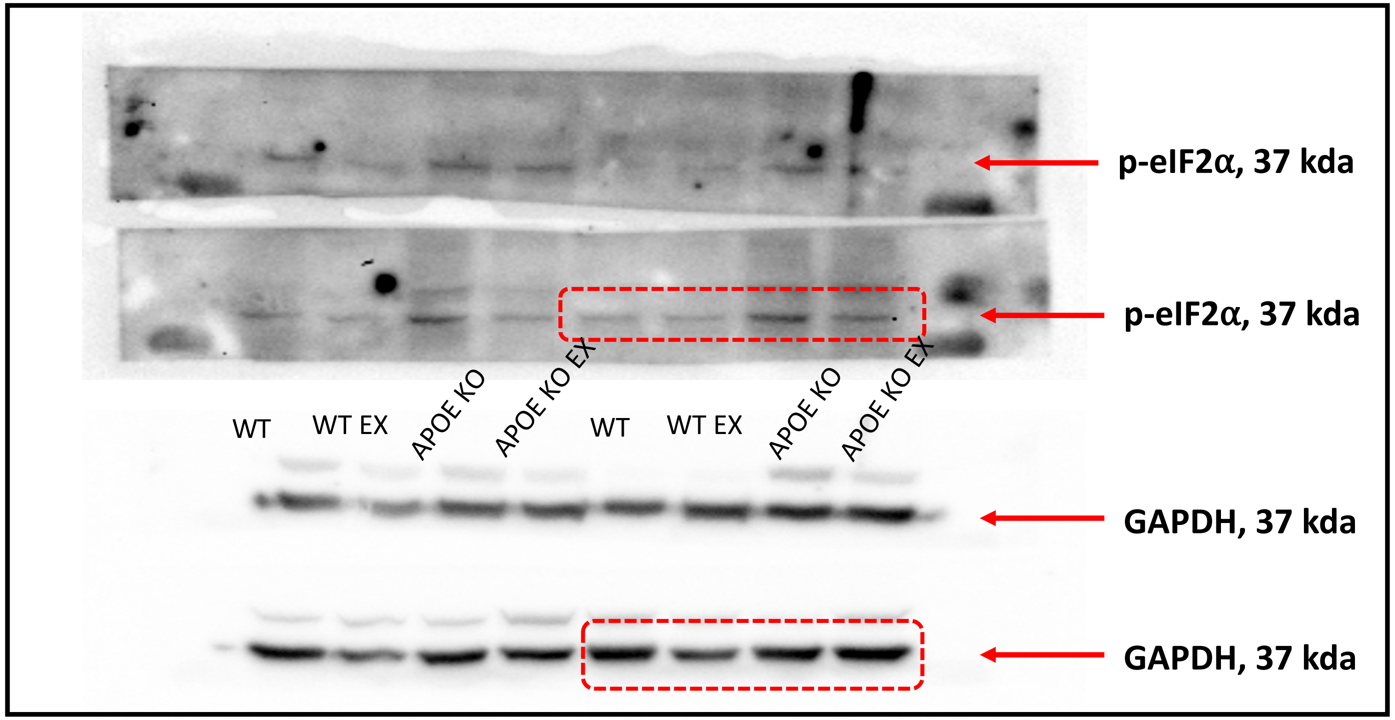

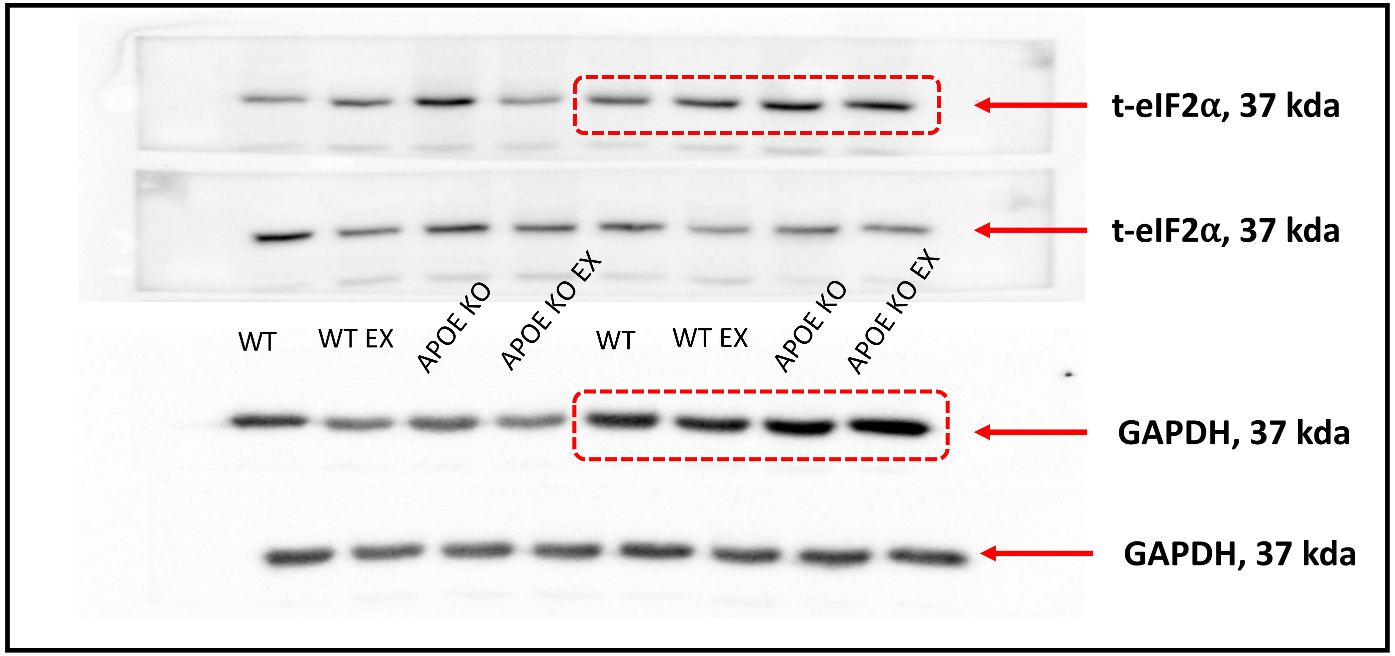

Supplementary Data
Representative Western blot images

Figure 2H- CHOP

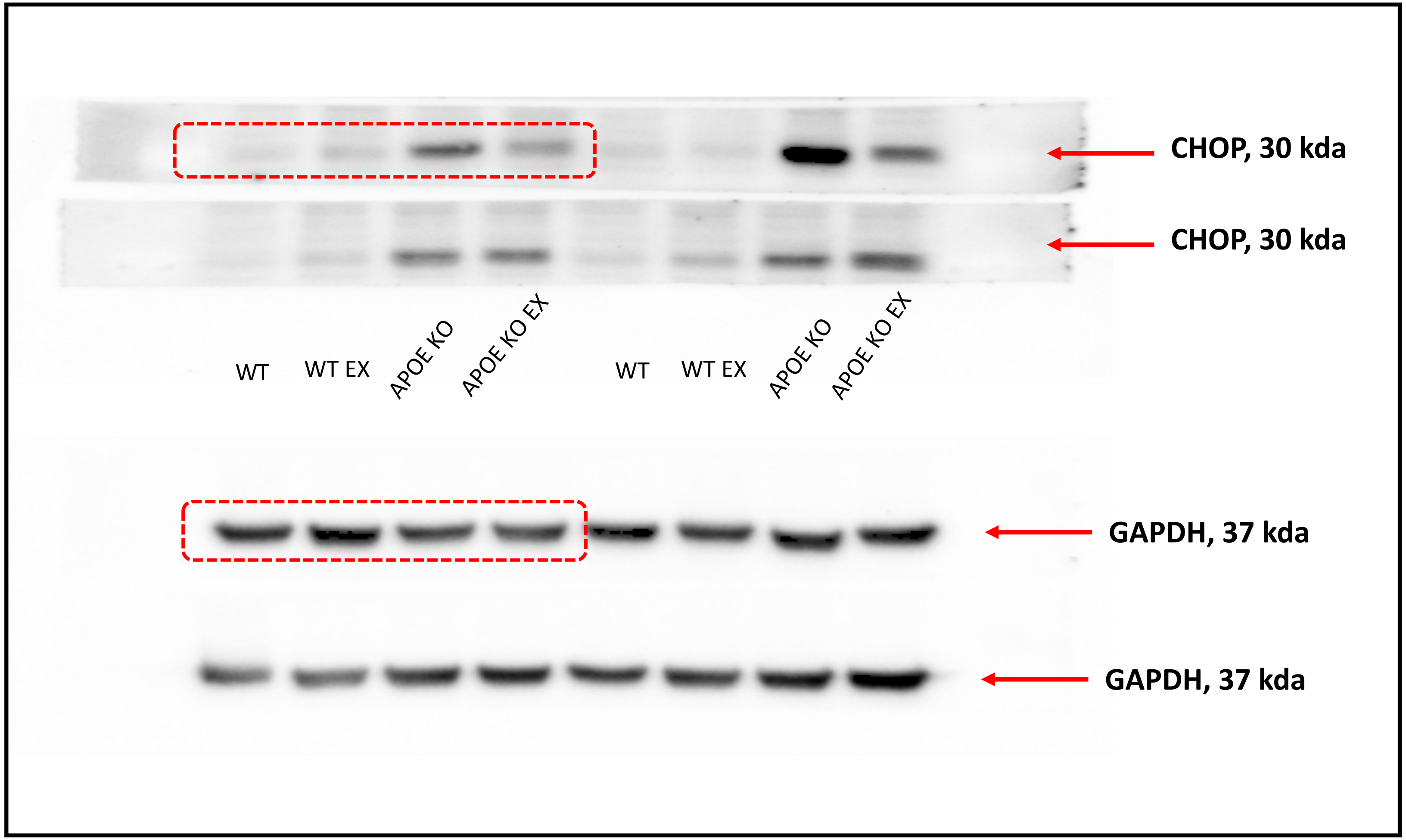

Supplementary Data- Representative Western blot images

Figure 2I- Bax

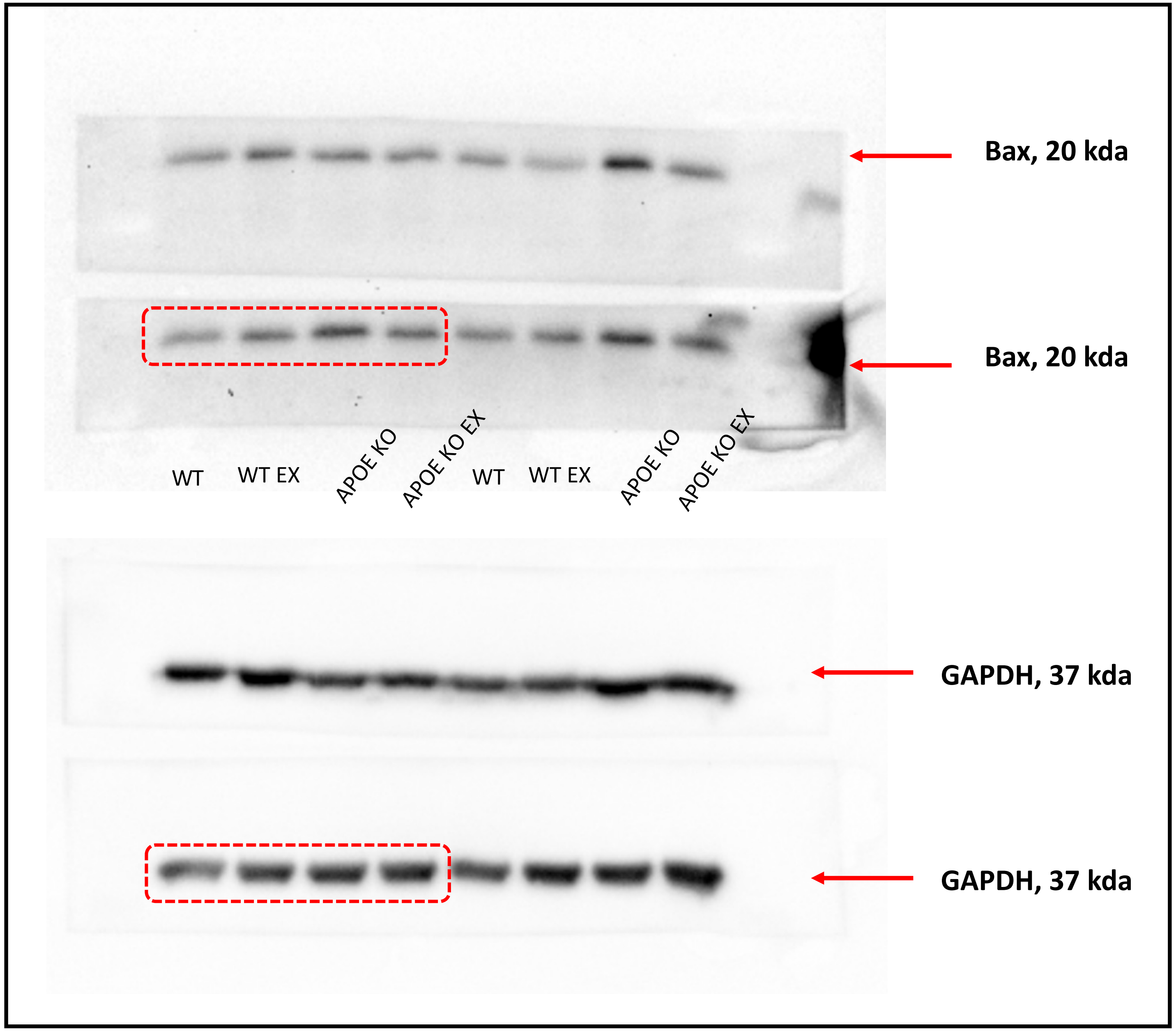

Supplementary Data
Representative Western blot images

Figure 3A- TXNIP

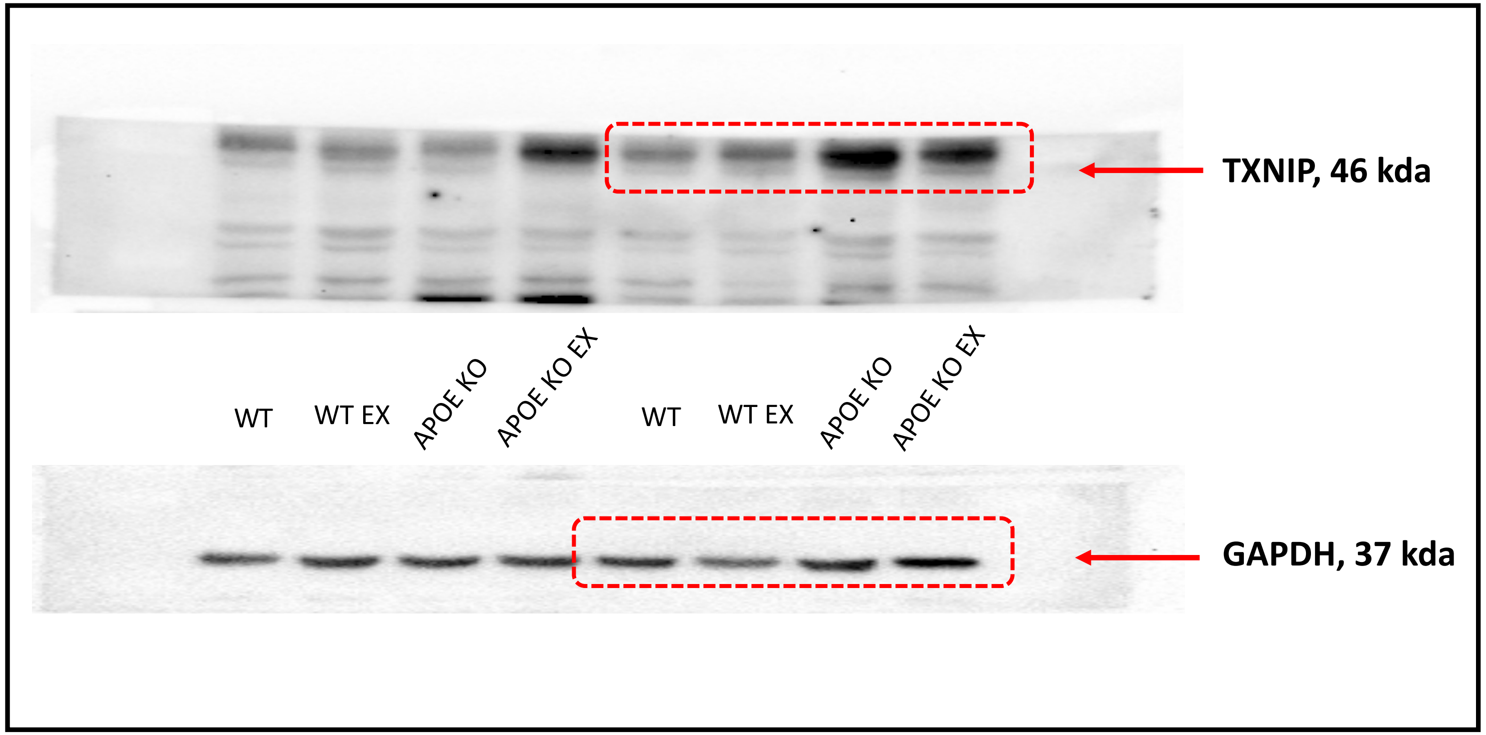

Supplementary Data- Representative Western blot images

Figure 3B- NLRP3

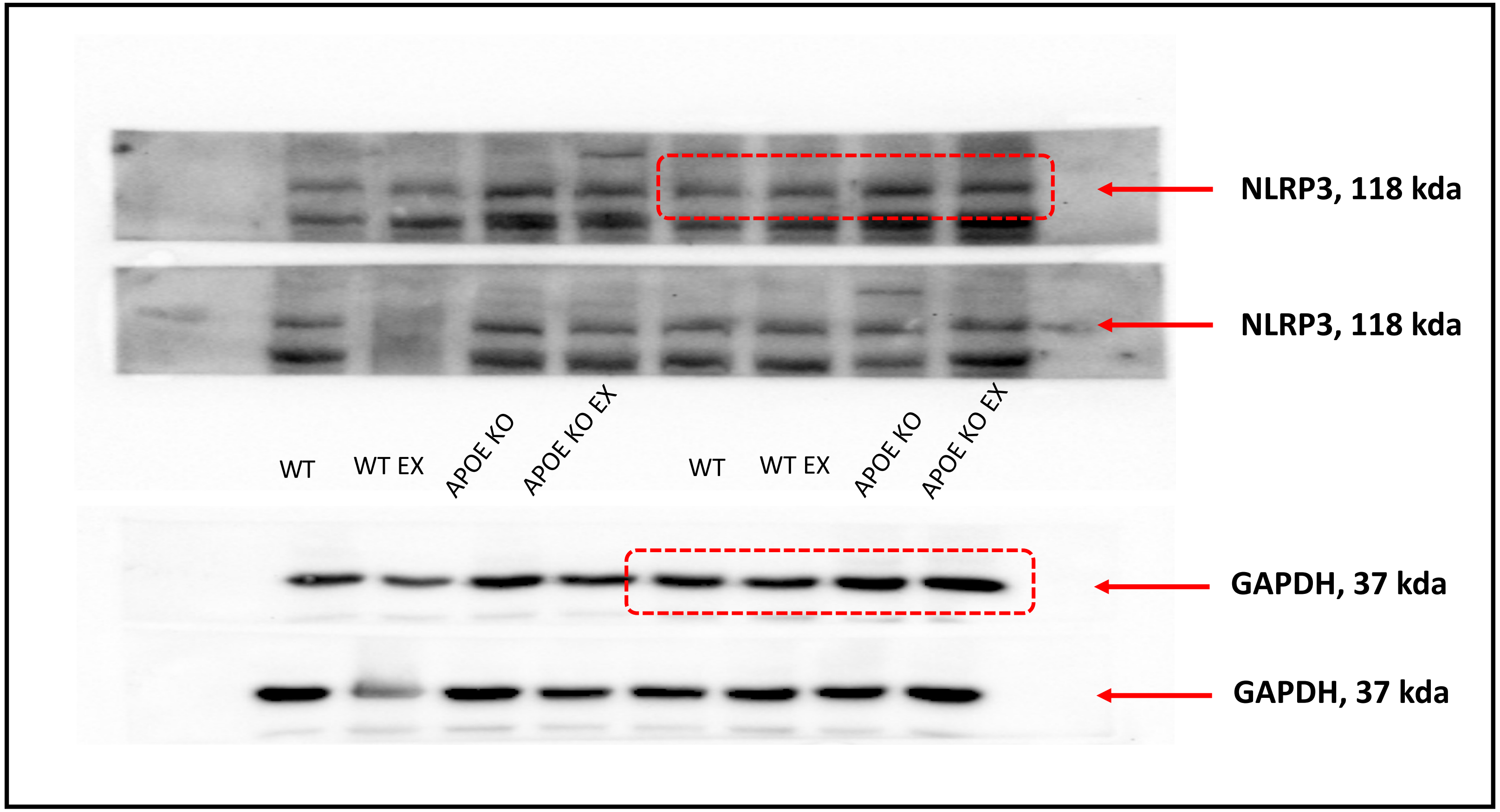

Supplementary Data- Representative Western blot images

Figure 3C & D- Procaspase-1 & P20

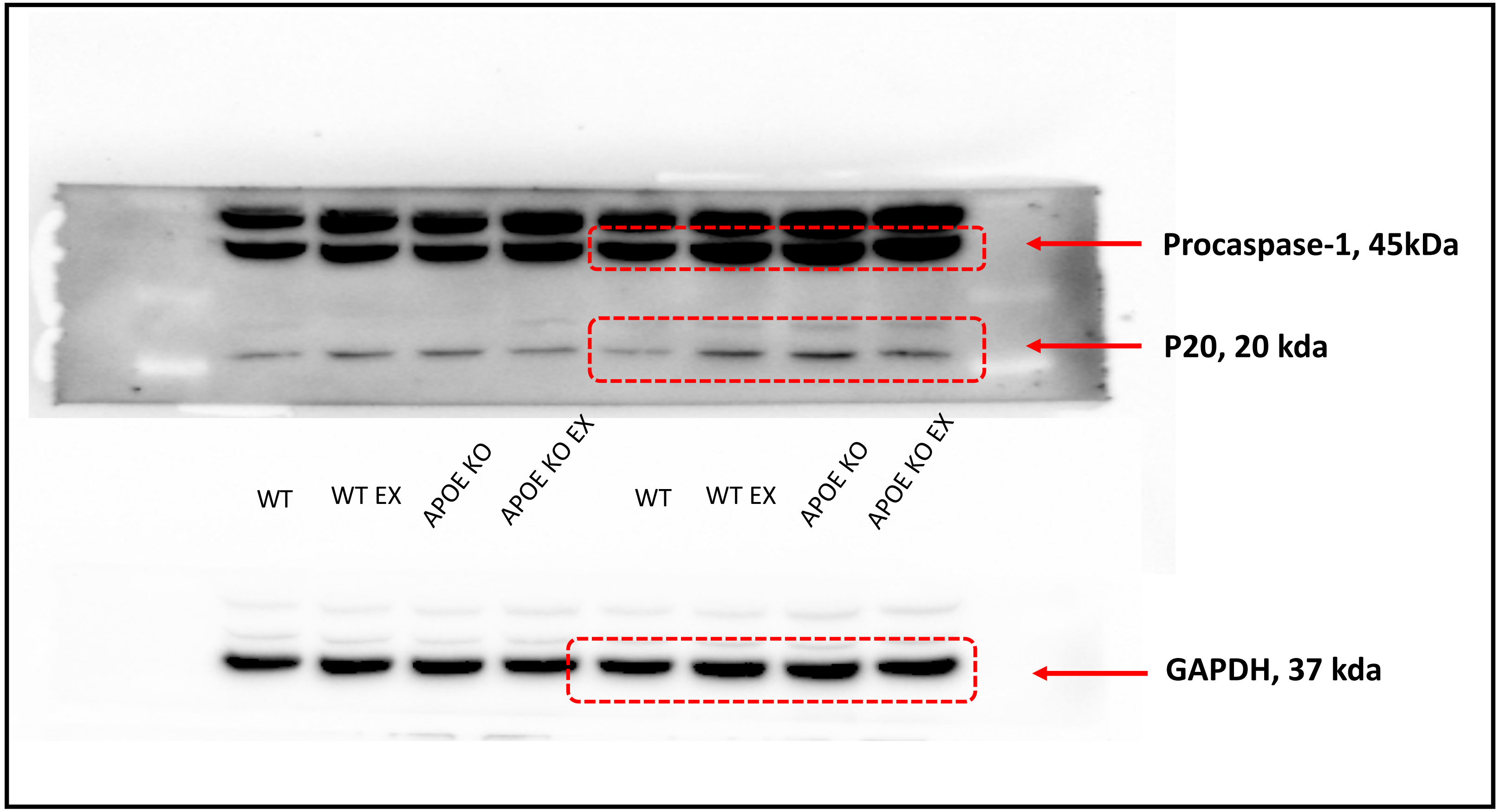

Supplementary Data- Representative Western blot images

Figure 3E- IL-1β

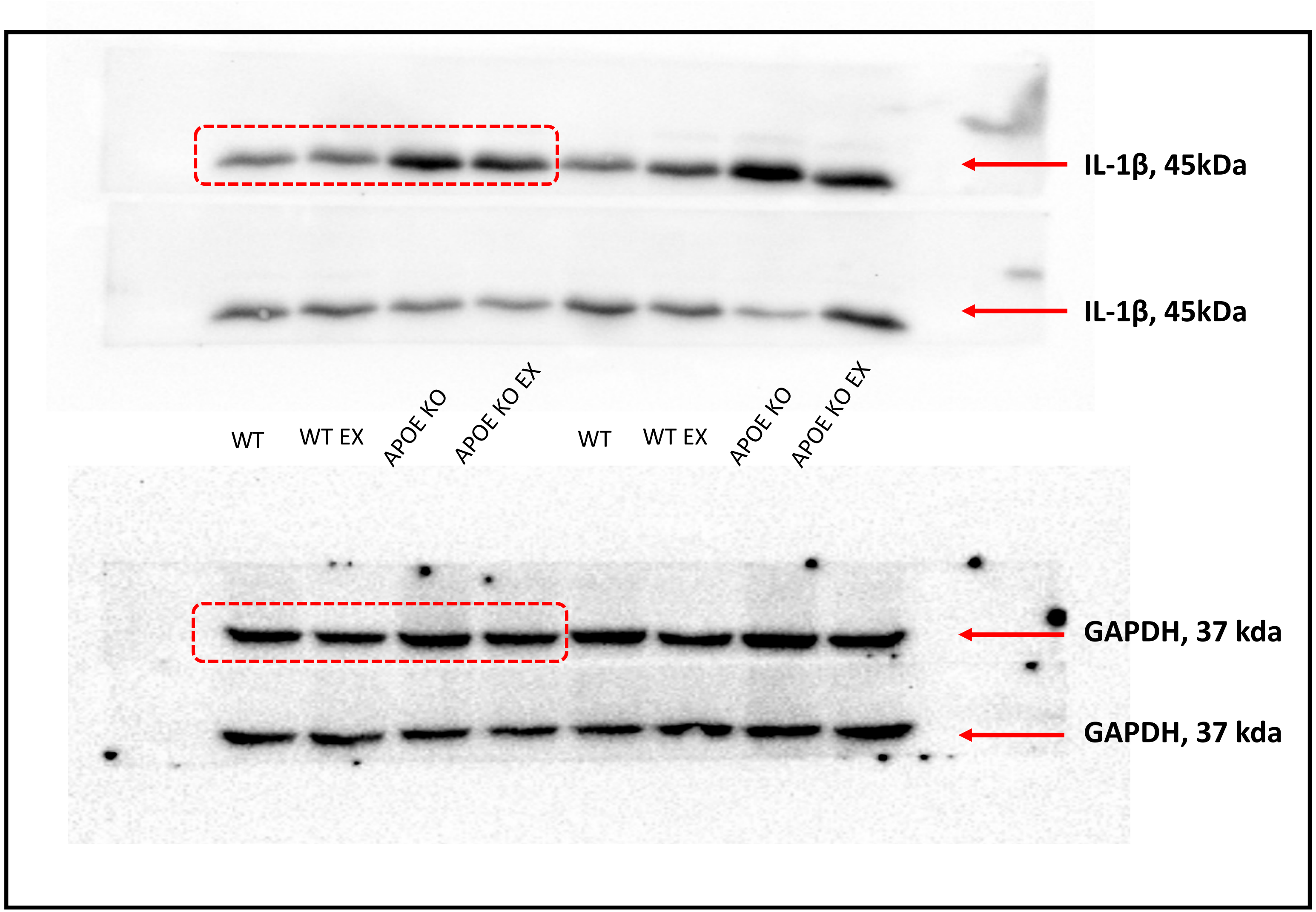

Supplementary Data- Representative Western blot images

Figure 4C- UCP2

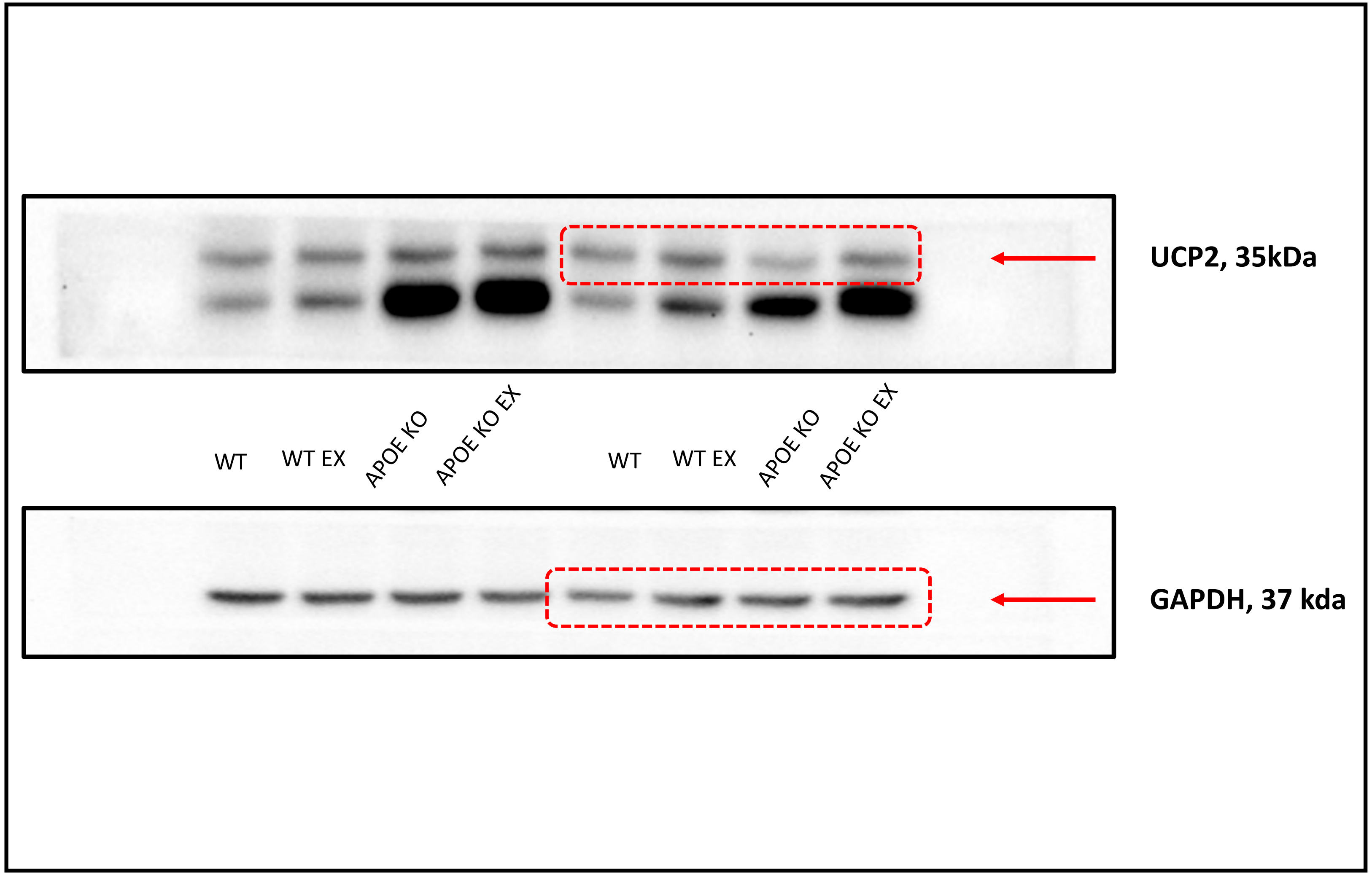

Supplement: Supplementary file 1 — Supplementary Information. [file 41598_2021_94944_MOESM1_ESM.pdf]
